# Incidence of and Risk Factors for Do-Not-Resuscitate Orders in Critically Ill Children: Insights From a Tertiary Care Center in Saudi Arabia

**DOI:** 10.1155/ccrp/9948312

**Published:** 2025-07-02

**Authors:** Tareq Alayed, Waad Al-Sowat, Abdullah Alturki, Fahad Aljofan, Moath Alabdulsalam, Tariq Alofisan, Raghad Alhuthil, Munirah Alshalawi, Mansour Alghamdi

**Affiliations:** ^1^Department of Critical Care Medicine, King Faisal Specialist Hospital and Research Center, Riyadh, Saudi Arabia; ^2^College of Medicine, Alfaisal University, Riyadh, Saudi Arabia; ^3^Department of Pediatrics, King Faisal Specialist Hospital and Research Center, Riyadh, Saudi Arabia

## Abstract

**Objectives:** To investigate the incidence and determinants of do-not-resuscitate (DNR) orders, as well as mortality-associated risk factors, in the pediatric intensive care unit (PICU) of a tertiary care center in Saudi Arabia.

**Design:** Retrospective cohort study.

**Setting:** The PICU at the King Faisal Specialist Hospital and Research Center, Riyadh, Saudi Arabia.

**Patients:** Patients aged 1 week to 14 years who were admitted to the PICU between January 2021 and December 2023.

**Interventions:** None.

**Measurements and Main Results:** Of the 3344 patients admitted to the PICU, 53.1% were male; the median age was 3 years (interquartile range: 0–8). The most common underlying conditions were neurological in 723 patients (21.6%), hematological/oncological in 463 (13.9%), and cardiovascular in 417 (12.5%). DNR orders were issued for 6.4% of admissions; among the 213 patients with DNR orders, 24 (11.3%) had a history of resuscitation before the DNR order. The mortality rate was significantly higher among patients with DNR orders (42.3%) compared to those without (1.3%; *p* < 0.001). Of all 3344 patients, 130 (3.9%) died; of these, 90 (69.2%) had DNR orders. Predictors of DNR status included male gender, hematological/oncological and cardiovascular diseases, bone marrow transplantation, respiratory distress, sepsis, seizures, bleeding, and need for mechanical ventilation (*p* < 0.05).

**Conclusions:** This study revealed a DNR order rate of 6.4% among all PICU admissions, with 69.2% of PICU deaths occurring in patients with DNR status. Further analysis is warranted to understand the factors influencing DNR decisions and their impact on patient outcomes.

## 1. Introduction

End-of-life decision-making for critically ill children in the pediatric intensive care unit (PICU) is a complicated and ethically challenging process that requires careful balancing of clinical assessment, the patient's clinical condition, and family preferences [1, 2].

Decisions regarding the initiation or withholding of life-sustaining treatments, including do-not-resuscitate (DNR) orders, are particularly sensitive in pediatric settings [3]. These decisions are affected by cultural, ethical, and religious factors that differ from one country to another.

In many countries, including Saudi Arabia, medically complex and technologically advanced interventions are often provided until the very end of life [4, 5]. Although these interventions can temporarily extend life, they can also extend suffering in patients with terminal conditions.

Recent progress in pediatric critical care points to the significance of involving palliative care and starting early, family-focused conversations about end-of-life decisions [1, 6]. These approaches encourage shared decision-making and aim to make sure that care plans are in line with the values and wishes of both the healthcare team and the family.

In Saudi Arabia, the Saudi Health Council developed the National Policy for DNR orders in 2017 to provide clear guidelines that respect Islamic law and cultural values [7]. This policy has clarified the process for DNR decisions and likely contributed to changes in clinical practice.

Previous data in our hospital (2007–2009) revealed that of the deceased patients, 51% (79 of 154) had DNR orders. However, no other relevant studies have been conducted more recently [8]. Therefore, our aims were to investigate the incidence and determinants of DNR orders, as well as mortality-associated risk factors, in the PICU of a tertiary care center in Saudi Arabia.

## 2. Materials and Methods

This retrospective cohort study included all pediatric patients aged > 1 week to 14 years who had been admitted to the PICU at the King Faisal Specialist Hospital and Research Center, Riyadh, Saudi Arabia, a private, nonprofit, tertiary care hospital. The PICU has approximately 20 beds and an average of 1000 admissions per year. In addition, most patients in the PICU are immunocompromised, with primary immunodeficiency, by chemotherapy, or by immunosuppressive therapy after organ transplantation.

The retrospective period for data collection was from January 2021 to December 2023. Patients' charts were reviewed electronically, and the extracted data included demographic information, medical history, PICU management, altered code status, PICU outcome, and length of hospitalization. All reported deaths occurred during the PICU admission; patients discharged to other settings for end-of-life care were not included in the mortality data.

### 2.1. DNR Protocol

All DNR codes in the reported cases were defined as withholding resuscitative measures in the event of cardiac or pulmonary arrest [7]. The decision requires consensus among three physicians, including at least one consultant, and applies to terminally ill patients, such as those with advanced cancer, irreversible organ failure, or conditions associated with an extremely poor prognosis.

DNR orders aim to avoid nonbeneficial, invasive interventions that may prolong suffering without improving outcomes. Instead, care shifts toward comfort-focused measures, prioritizing the patient's dignity and quality of life in alignment with family and medical team discussion.

### 2.2. Data Analysis

To analyze the data, we used Stata Version 18 (StataCorp, LLC, College Station, TX, USA). Data for categorical variables were calculated as frequencies (*n*s) and percentages. Data for continuous variables were calculated as medians and interquartile ranges. We performed Mann–Whitney *U* and chi-square tests to investigate DNR order and mortality-related risk factors, and we used a logistic regression model to perform multivariable analysis. A *p* value of < 0.05 was considered significant. Multicollinearity among independent variables was assessed using the variance inflation factor (VIF), and all variables had VIF values less than five, confirming no significant multicollinearity.

## 3. Results

This study included data from 3344 patients admitted to the PICU between 2021 and 2023. Boys accounted for 53.1% (*n* = 1775) and girls 46.9% (*n* = 1569). The median age at admission was 3 years (interquartile range, 0–8). The most common underlying diseases were neurological (21.6%), hematological/oncological (13.9%), and cardiovascular (12.5%) disorders. Other notable diagnoses included hepatological (12.1%), nephrological (8.0%), and pulmonological (7.8%) diseases. Additionally, 10.9% of patients had undergone solid organ transplantation, and 4.4% had undergone bone marrow transplantation (Table 1).

Patients were primarily admitted to the PICU for postoperative care (43.5%) and management of respiratory distress (20.5%). In terms of interventions, 30.4% required mechanical ventilation, 26.7% received high-flow nasal cannula (HFNC) therapy, and 19.9% had central venous catheters (Table 1).

The incidence of DNR orders was 6.4% (213/3344), corresponding to 14.09 DNR orders per 1000 PICU days. Among them, 11.3% (24/213) had undergone previous resuscitation attempts prior to the DNR order. The median PICU stay was 2 days (IQR: 1–5), and the overall PICU mortality rate was 3.9% (130/3344). Most deaths occurred in patients with DNR orders (69.2% [90/130]), with a mortality rate of 42.3% (90/213) compared to 1.3% (40/3091) in patients without DNR orders (*p* < 0.001) (Table 1).

### 3.1. Factors Associated With DNR Orders

The multivariable analysis identified several significant predictors of DNR status: cardiovascular disorder (OR = 1.69, 95% CI: 1.09–2.62, *p*=0.019), solid organ transplantation, which was associated with lower odds of DNR orders (OR = 0.24, 95% CI: 0.08–0.70, *p*=0.009), respiratory distress as reason for PICU admission (OR = 2.97, 95% CI: 1.94–4.55, *p* < 0.001), sepsis (OR = 2.04, 95% CI: 1.13–3.67, *p*=0.018), seizure disorder (OR = 4.31, 95% CI: 2.20–8.46, *p* < 0.001), bleeding (OR = 5.78, 95% CI: 2.25–14.85, *p* < 0.001), mechanical ventilation (OR = 1.97, 95% CI: 1.33–2.94, *p*=0.001), HFOV (OR = 2.28, 95% CI: 1.13–4.60, *p*=0.021), CRRT (OR = 2.42, 95% CI: 1.10–5.30, *p*=0.028), inotropic support (OR = 1.88, 95% CI: 1.20–2.94, *p*=0.006), and palliative care involvement (OR = 7.27, 95% CI: 4.73–11.17, *p* < 0.001; Table 2).

### 3.2. Baseline Characteristics of Patients Who Died in the PICU

Among the 130 patients who died during their PICU stay, 90 (69.2%) had documented DNR orders while 40 (30.8%) did not. The median age of the overall deceased cohort was 2 years (IQR: 0–6), and 74 (56.9%) were male. Hematological/oncological disorders were the most common comorbidities, present in 31.5% of patients, followed by cardiovascular disorders (20.8%) and bone marrow transplantation (16.9%). A majority required mechanical ventilation (81.5%), and 26.2% received HFOV. Palliative care involvement was documented in 22.3% of the cases (Table 3).

### 3.3. Factors Associated With Mortality

In the univariable analysis, none of the demographic or medical history variables were significantly associated with having a DNR order among deceased patients. However, respiratory distress as a reason for PICU admission was more common among patients with DNR orders compared to those without (46.7% vs. 27.5%, *p*=0.040). Additionally, referral to palliative care was significantly more frequent among patients who died with DNR orders (27.8% vs. 10.0%, *p*=0.025). In the multivariable analysis, only referral to palliative care remained significantly associated with DNR status among deceased patients (OR = 3.31, 95% CI: 1.06–10.39, *p*=0.040; Table 3).

## 4. Discussion

In this study, we explored the incidence of and factors underlying DNR orders, as well as deaths related to DNR, in the PICU of a Saudi tertiary care center. We found that DNR orders were present in 6.4% of all patients admitted to the PICU and in 69% of all patients who died in the PICU.

Aletreby et al. reported that 14.9% of all ICU discharges were patients who died in the ICU with a documented DNR order, representing 63% of all ICU deaths [9], whereas other investigators reported DNR rates ranging from 6.4% to 9% [10–12]. In addition, Zingmond and Wenger found that DNR orders accounted for up to 15% of all ICU discharges in hospitals with 300 or more beds [13]. Thus, our DNR incidence was within the range reported in other studies; however, the populations of those studies were mainly adult patients, and more investigation among pediatric populations is required.

Among all deceased patients, 69.5% had documented DNR orders, reflecting the increasing recognition and implementation of end-of-life care decisions in the PICU. While the overall PICU mortality rate was 3.9%, the mortality rate among patients with DNR orders (41%) was significantly higher than that among patients without DNR orders (1.3%; *p* < 0.001). Similarly, in a single-center study, Fuchs et al. analyzed the DNR orders in adult ICUs in Israel, involving 19,007 patients from 2001 to 2008 [14]. Of those patients, 1239 (6.5%) had a DNR order upon ICU admission and survived for 48 h in the ICU. In comparing these patients with 2402 patients who had no DNR orders, Fuchs et al. found that both the 28-day and 1-year mortality rates were significantly higher among patients with DNR orders than among those without (33.9% vs. 18.4% and 60.7% vs. 40.2%, respectively; *p* < 0.001) [14].

Compared to a previous study in our institution (2007–2009), where 51% of PICU deaths were associated with a DNR order [8], we observed a rise to nearly 69.5% in 2021–2023. This change may reflect growing recognition of the role of DNR orders in honoring patient dignity, minimizing suffering, and avoiding nonbeneficial interventions at the end of life [5]. The 2017 Saudi National Policy for DNR orders, which provides a standardized framework aligning with Islamic law and cultural values, may have contributed to this trend by guiding multidisciplinary decision-making and emphasizing palliative care and patient-centered practices [7]. However, further research is needed to elucidate the drivers of this trend—whether cultural, institutional, or evidence-based—and its impact on outcomes.

As for factors associated with DNR orders, the univariable analysis found an association between male gender and the existence of DNR orders; in contrast, other studies conducted among adult populations revealed that female gender was more associated with DNR orders [15, 16]. The association between male gender and DNR status is novel and may reflect cultural or familial dynamics, which should be explored in future studies.

Furthermore, we identified several clinical conditions significantly associated with DNR orders in the multivariable analysis. Patients with cardiovascular diseases, respiratory distress, sepsis, seizure disorders, and bleeding were more likely to have DNR orders. This finding is consistent with those of prior research that indicated that severe, progressive, or incurable conditions often lead to DNR decisions [6, 17].

The need for intensive interventions, such as mechanical ventilation, HFOV, inotropic support, and CRRT, were also predictors of the existence of DNR orders. These interventions are typically performed for extremely severe illness and indicate a poorer prognosis [1]. Additionally, the association between palliative care involvement and DNR status underscores the team's critical role in facilitating family-centered decision-making [1, 2]. Although palliative care was involved in only a subset of cases, its strong association with DNR orders may reflect its targeted involvement in patients with a poor prognosis, which points to the need for earlier palliative care integration in the PICU. Their integration may explain part of the observed rise in DNR orders, as palliative teams prioritize symptom management and align care with patient/family values.

One notable finding was the negative association between solid organ transplantation and DNR status; the reason could be that patients who undergo these transplantations may receive more aggressive care than those with other conditions. However, further investigation is necessary to support this finding.

While our study did not assess family perspectives, cultural and religious attitudes are known to play a central role in DNR decisions in the Middle East. For example, Sabouneh et al. found that only 34% of families consented to DNR orders in a pediatric ICU, with many preferring a “soft” resuscitation approach. This practice, as described by the authors, involves administering a few doses of epinephrine without performing chest compressions. This form of limited resuscitation may help families who are emotionally or culturally hesitant to consent to a formal DNR order [18]. On a policy level, shifts in legal frameworks can reshape practice. In South Korea, the Life-Sustaining Treatment Decision Act led to a significant drop in DNR orders and an increase in formal withdrawal of life support, highlighting the impact of structured legal pathways [19].

Globally, approaches to pediatric DNR decisions vary. In the United States, decisions are often shaped by early efforts at shared decision-making with empathy, where all options are carefully explored between care teams and families [20]. While in countries such as Indonesia, the lack of clear legal guidance continues to challenge end-of-life care for children [21]. These international perspectives underscore the importance of aligning DNR practices with legal, ethical, and cultural norms, especially in pediatric care, where family-centered decision-making is paramount.

On the other hand, the Saudi National Policy for DNR orders (2017) requires DNR orders to be made by three physicians (including at least one consultant) for terminally ill patients when resuscitation is deemed futile. It also mandates regular review of DNR status and emphasizes that these orders should not affect palliative care or patient comfort [7]. This policy reflects a balanced, culturally sensitive approach that integrates medical ethics with religious and societal values.

### 4.1. Limitations

Our study had several limitations, including its retrospective design, which may be subject to biases inherent in medical record reviews. Moreover, our findings are specific to a single tertiary care center in Saudi Arabia; thus, the generalizability of those findings to other settings or regions might be limited. Furthermore, the study's findings are restricted to DNR orders within critically ill pediatric patients and did not capture family attitudes or physician perspectives, which are essential components of the decision-making process.

## 5. Conclusions

Our study provides valuable insight into the incidence and characteristics of DNR orders in a pediatric intensive care setting in Saudi Arabia. With a DNR rate of 6.4% for all PICU admissions and a rate of 69.5% among PICU deaths, these findings reflect evolving practices in pediatric end-of-life care. Notably, 11% of those with DNR had prior resuscitation events. Clinical conditions such as cardiovascular disease, respiratory distress, sepsis, seizures, and bleeding were strongly associated with DNR decisions, along with the use of intensive life-sustaining therapies. The findings underscore the importance of early, culturally appropriate discussion and the role of palliative care in supporting families and healthcare providers in making compassionate, ethically sound end-of-life decisions.

## Figures and Tables

**Table 1:** Characteristics of study participants (*N* = 3344).

Characteristic	*n* (%)^a^
Gender	
Male	1775 (53.1)
Female	1569 (46.9)
Age (years), median [IQR]	3 [0–8]
Underlying disease	
Neurological	723 (21.6)
Hematological/oncological	463 (13.9)
Cardiovascular	417 (12.5)
Hepatological	405 (12.1)
Nephrological	267 (8.0)
Pulmonological	260 (7.8)
Gastroenterological	203 (6.1)
Genetic	148 (4.4)
Allergy/immunological	141 (4.2)
Endocrinological	130 (4.0)
Others	187 (5.6)
Type of transplantation	
Solid organ	363 (10.9)
Bone marrow	146 (4.4)
Reason for PICU admission	
Postoperative care	1456 (43.5)
Respiratory distress	684 (20.5)
Tube/catheter insertion	229 (6.9)
Decreased level of consciousness	230 (6.9)
Sepsis	209 (6.3)
Cardiac conduction disorders	126 (3.8)
Seizure	97 (2.9)
Brain injury	86 (2.6)
Bleeding	42 (1.3)
Metabolic crisis	40 (1.2)
Hepatic encephalopathy	37 (1.1)
Liver failure	30 (0.9)
Others	78 (2.3)
Interventions in the PICU	
Mechanical ventilation	1017 (30.4)
High-flow nasal cannula	893 (26.7)
CVC insertion	666 (19.9)
Inotropic support	389 (11.6)
Noninvasive ventilation	182 (5.4)
Abdominal drain insertion	103 (3.1)
Chest tube insertion	99 (3.0)
Administration of Factor VII	94 (2.8)
HFOV	65 (1.9)
CRRT	60 (1.8)
Palliative care	156 (4.7)
Code status	
DNR	213 (6.4)
Full code (no DNR)	3131 (93.6)
A history of resuscitation before the DNR order	24/213 (11.3)
PICU stay (days), median [IQR]	2 [1–5]
Mortality among patients with DNR orders^b^	90/213 (42.3)
Mortality among patients without DNR orders (failed CPR)^b^	40/3091 (1.3)
Overall PICU mortality	130 (3.9)

*Note:* DNR = do-not-resuscitate order.

Abbreviations: CPR = cardiopulmonary resuscitation, CRRT = continuous renal replacement therapy, CVC = central venous catheter, HFOV = high-frequency oscillatory ventilation, and PICU = pediatric intensive care unit.

^a^Except where noted, all data in this column are *n*s (percentages in parentheses).

^b^Mortality rates were significantly higher among patients with DNR orders than those among patients without DNR orders (chi-square *p* < 0.001).

**Table 2:** Factors associated with DNR orders (*N* = 3344).

Factors	Univariable analysis^a^			Multivariable analysis^b^	
	DNR (*n* = 213)	Non-DNR (*n* = 3131)	*p*	OR [95% CI]	*p*
Demographics					
Male gender	130 (61.0)	1645 (52.5)	0.016^c^	1.33 [0.96–1.84]	0.086
Female gender	83 (39.0)	1486 (47.5)			
Age (years), median [IQR]	3 [0–6]	3 [0–8]	0.267	—	—
Medical history					
Hematological/oncological disease	47 (22.1)	416 (13.3)	<0.001^c^	1.31[0.83–2.09]	0.248
Cardiovascular disease	42 (19.7)	375 (12.9)	0.001^c^	1.69 [1.09–2.62]	0.019^c^
Solid organ transplantation	4 (1.9)	359 (11.8)	<0.001^c^	0.24 [0.08–0.70]	0.009^c^
Bone marrow transplantation	21 (9.9)	125 (4.0)	<0.001^c^	1.04 [0.52–2.07]	0.912
Reason for PICU admission					
Respiratory distress	91 (42.7)	593 (18.9)	<0.001^c^	2.97 [1.94–4.55]	<0.001^c^
Hepatic encephalopathy	2 (0.9)	35 (1.1)	1.000	—	—
Sepsis	24 (11.3)	185 (5.9)	0.002^c^	2.04 [1.13–3.67]	0.018^c^
Seizure disorder	14 (6.6)	83 (2.7)	0.001^c^	4.31 [2.20–8.46]	<0.001^c^
Bleeding	11 (5.2)	31 (1.0)	<0.001^c^	5.78 [2.25–14.85]	<0.001^c^
Liver failure	2 (0.9)	28 (0.9)	0.717	—	—
PICU interventions					
Mechanical ventilation	130 (61.0)	887 (28.4)	<0.001^c^	1.97 [1.33–2.94]	0.001^c^
High-flow nasal cannula	102 (47.9)	791 (25.3)	<0.001^c^	0.95 [0.64–1.42]	0.817
CVC insertion	104 (48.8)	562 (18.0)	<0.001^c^	1.47 [0.98–2.21]	0.065
Inotropic support	81 (38.0)	308 (9.8)	<0.001^c^	1.88 [1.20–2.94]	0.006^c^
Noninvasive ventilation	34 (16.0)	148 (4.7)	<0.001^c^	1.34 [0.78–2.30]	0.282
Abdominal drain insertion	14 (6.6)	89 (2.8)	0.006^c^	0.91 [0.37–2.24]	0.830
Chest tube insertion	12 (5.6)	87 (2.8)	0.032^c^	0.49 [0.20–1.20]	0.118
Administration of Factor VII	37 (17.4)	57 (1.8)	<0.001^c^	1.89 [0.98–3.64]	0.056
HFOV	28 (13.2)	37 (1.2)	<0.001^c^	2.28 [1.13–4.60]	0.021^c^
CRRT	19 (8.9)	41 (1.3)	<0.001^c^	2.42 [1.10–5.30]	0.028^c^
Palliative care	68 (31.9)	88 (2.8)	<0.001^c^	7.27 [4.73–11.17]	<0.001^c^
PICU stay (days), median [IQR]	6 [3–12]	2 [1–4]	<0.001^c^	1.00 [0.99–1.02]	0.459

*Note:* Data presented as frequency (%) or median [interquartile range]. DNR = do-not-resuscitate order. The bold values in Table 2 indicate statistically significant results (*p* < 0.05), as mentioned in the methods section.

Abbreviations: CRRT = continuous renal replacement therapy, CVC = central venous catheter, HFOV = high-frequency oscillatory ventilation, and PICU = pediatric intensive care unit.

^a^The chi-square and Mann–Whitney *U* tests were used to perform univariable analysis.

^b^The logistic regression model was used to perform the multivariable analysis.

^c^Significant *p* value.

**Table 3:** Factors associated with patients who died with and without DNR orders (*N* = 130).

Factors	Total (*n* = 130)	Univariable analysis^a^			Multivariable analysis^b^	
		Died with DNR (*n* = 90)	Died without DNR (*n* = 40)	*p*	OR [95% CI]	*p*
Demographics						
Male gender	74 (56.9)	55 (61.1)	19 (47.5)	0.148	—	—
Female gender	56 (43.1)	35 (38.9)	21 (52.5)		—	—
Age (years), median [IQR]	2 [0–6]	2 [0–7]	2 [1–5.5]	0.961	—	—
Medical history						
Hematological/oncological disorder	41 (31.5)	30 (33.3)	11 (27.5)	0.509	—	—
Cardiovascular disorder	27 (20.8)	22 (24.4)	5 (12.5)	0.121	—	—
Solid organ transplantation	6 (4.6)	4 (4.4)	2 (5.0)	1.000	—	—
Bone marrow transplantation	22 (16.9)	16 (17.8)	6 (15.0)	0.697	—	—
Reason for PICU admission						
Respiratory distress	53 (40.8)	42 (46.7)	11 (27.5)	0.040^c^	2.21 [0.97–5.04]	0.058
Hepatic encephalopathy	3 (2.3)	2 (2.2)	1 (2.5)	1.000	—	—
Sepsis	21 (16.2)	13 (14.4)	8 (20.0)	0.427	—	—
Bleeding	10 (7.7)	7 (7.8)	3 (7.5)	1.000	—	—
Liver failure	4 (3.1)	2 (2.2)	2 (5.0)	0.586	—	—
PICU interventions						
Mechanical ventilation	106 (81.5)	73 (81.1)	33 (82.5)	0.851	—	—
High-flow nasal cannula	55 (42.3)	39 (43.3)	16 (40.0)	0.723	—	—
CVC insertion	83 (63.9)	60 (66.7)	23 (57.5)	0.330	—	—
Inotropic support	80 (61.5)	54 (60.0)	26 (65.0)	0.589	—	—
Noninvasive ventilation	20 (15.4)	14 (15.6)	6 (15.0)	0.935	—	—
Abdominal drain insertion	15 (11.5)	11 (12.2)	4 (10.0)	1.000	—	—
Chest tube insertion	18 (13.9)	12 (13.3)	6 (15.0)	0.789	—	—
Administration of Factor VII	42 (32.3)	28 (31.1)	14 (35.0)	0.662	—	—
HFOV	34 (26.2)	25 (27.8)	9 (22.5)	0.527	—	—
CRRT	24 (18.5)	18 (20.0)	6 (15.0)	0.627	—	—
Palliative care	29 (22.3)	25 (27.8)	4 (10.0)	0.025^c^	3.31 [1.06–10.39]	0.040^c^
PICU stay (days), median [IQR]	7.5 [4–13]	8 [5–13]	6 [2–13.5]	0.159	—	—

*Note:* Data presented as frequency (%) or median [interquartile range]. DNR = do-not-resuscitate order. The bold values in Table 3 denote statistically significant results (*p* < 0.05), as described in the methods section.

Abbreviations: CRRT = continuous renal replacement therapy, CVC = central venous catheter, HFOV = high-frequency oscillatory ventilation, and PICU = pediatric intensive care unit.

^a^The chi-square and Mann–Whitney *U* tests were used to perform univariable analysis.

^b^The logistic regression model was used to perform the multivariable analysis.

^c^Significant *p* value.

## Data Availability

The data supporting this study are available upon reasonable request from the corresponding author.
